# Quantitative assessment of cardiovascular autonomic impairment in cancer survivors: a single center case series

**DOI:** 10.1186/s40959-020-00065-9

**Published:** 2020-07-28

**Authors:** Benjamin Noor, Shannel Akhavan, Michael Leuchter, Eric H. Yang, Olujimi A. Ajijola

**Affiliations:** 1 Division of Internal Medicine, Department of Medicine, University of California at Los Angeles, Los Angeles, CA USA; 2 UCLA Cardio-Oncology Program and Division of Cardiology, Department of Medicine, University of California at Los Angeles, Los Angeles, CA USA; 3 UCLA Cardiac Arrhythmia and Neurocardiology Research Center, David Geffen School of Medicine at UCLA, University of California at Los Angeles, 100 Medical Plaza, Suite 660, Westwood Blvd, Los Angeles, CA 90095-1679 USA

**Keywords:** Cardiovascular autonomic dysfunction, Autonomic reflex testing, Syncope, Palpitations, Postural orthostatic tachycardia, Orthostatic hypotension, Inappropriate sinus tachycardiac, Anthracyclines, Vinca alkaloids, Alkylating agents

## Abstract

**Background:**

Cardiovascular autonomic dysfunction in cancer survivors is poorly understood.

**Objectives:**

To better characterize the clinical characteristics and types of autonomic dysfunction in this population.

**Methods:**

A retrospective analysis of cancer survivors within an academic cardio-oncology program referred for suspected autonomic dysfunction was performed. Autonomic reflex testing of adrenergic, cardiovagal, and sudomotor function was done. Autonomic impairment was graded on severity based on the Composite Autonomic Severity Score system. Patients with pre-existing autonomic dysfunction prior to their cancer diagnosis were excluded.

**Results:**

Of approximately 282 total patients in the UCLA Cardio-Oncology program, 24 were referred for suspected autonomic dysfunction and met the inclusion criteria. 22 had autonomic impairment on autonomic reflex testing. Eight patients were female, and the mean age at time of autonomic testing was 51.3 years. The average duration from cancer diagnosis to autonomic testing was 10.3 years. The reasons for referral included dizziness, tachycardia, palpitations, and syncope. The majority of patients (75%) had hematologic disorders. The most common chemotherapies administered were vinca alkaloids (54.2%), alkylating agents (66.7%), and anthracyclines (54.2%). Most patients received radiation to the thorax (66.7%) and neck (53.3%). Eleven patients had mild autonomic impairment, 7 had moderate, and 4 had severe autonomic impairment. Dysfunction was commonly present in the sympathetic and parasympathetic branches, but most pronounced in the sympathetic system. The majority of patients were diagnosed with orthostatic hypotension (50%), inappropriate sinus tachycardia (20.8%), and postural orthostatic tachycardia syndrome (12.5%) and had subjective improvement with treatment.

**Conclusion:**

Cardiovascular autonomic dysfunction occurs in cancer survivors, and commonly affects both the sympathetic and parasympathetic systems. Symptom recognition in patients should prompt autonomic testing and treatment where appropriate.

## Background

Long term cancer survival rates have increased largely due to the improvements in detection and treatment of malignancies, increased detection of less malignant cancers, and advancements in the management of non-cancer-related health conditions. The five-year relative survival rate for all cancers has increased from 49% in 1975–1977 to 69% in 2008–2014 [1]. By 2030, the population of cancer survivors in the United States is estimated to be at least 22.1 million [2]. Because of prolonged survival, long-term sequalae of cancer and cancer-directed treatments have increased in prevalence [3]. One known, but poorly understood, comorbidity in cancer survivors is cardiovascular autonomic dysfunction [4–6].

Cardiovascular autonomic impairment has been detected in 52–81% of patients with advanced cancer [7–10]. Due to an imbalance in the physiologic interplay between the sympathetic and parasympathetic systems, patients may experience symptoms such tachycardia, syncope, palpitations, or dizziness. Proposed acute and chronic mechanisms of autonomic injury in cancer survivors include tumor compression or invasion of autonomic nerves, paraneoplastic effects, side effects of chemoradiation, and deconditioning [5, 9, 11, 12]. Autonomic dysfunction is speculated to be an early marker of cardiovascular risk in cancer patients and is associated with an increase all-cause mortality [4, 13]. This highlights the potential survival implications of understanding this condition, in addition to managing the debilitating symptoms patients with autonomic dysfunction often face. Most studies on cardiovascular autonomic dysfunction in cancer survivors focus on the presence or absence of impairment and occasionally grade the severity of autonomic impairment [7–10, 14]. However, the subtypes and severity of autonomic dysfunction, and associations with specific treatment agents remain poorly understood.

## Methods

### Study aim

The aim of this study is to characterize and quantify the type and severity of cardiovascular autonomic dysfunction in cancer survivors in a cardio-oncology program, and identify associations between malignancies or treatments with cardiovascular autonomic dysfunction.

### Study design and setting

A retrospective analysis of cancer survivors within an academic cardio-oncology program referred for autonomic dysfunction was performed. The electronic medical records were reviewed from January 1st 2010 until December 15th 2019, and study data were extracted. The UCLA institutional review board approval was obtained.

### Patient selection

Patients at least 18 years of age in the cardio-oncology program with a history of malignancy, chemotherapy, or radiation therapy who were referred to the University of California at Los Angeles (UCLA) autonomic testing lab were included in the study. Patients were excluded if they did not undergo autonomic testing or if they had known cardiovascular autonomic dysfunction prior to being diagnosed with cancer.

### Data collection

Patient demographics, symptoms, medical history, cancer-related treatments, echocardiography, cardiodiagnostics, autonomic function test results, and subjective response to autonomic dysfunction directed treatments were collected. Malignancies were classified based on the anatomic location where the majority of the disease was located. In the case of leukemia or diffuse lymphoma, where a particular anatomic location could not be specified, location of disease was categorized as blood and bone marrow. Ventricular function was assessed by echocardiography using left ventricular ejection fraction (LVEF) and myocardial strain. Strain was measured by quantifying global longitudinal strain. Abnormal strain values were based on the age-and-gender adjusted normative values of the software used for interpretation.

### Autonomic reflex testing protocol

Autonomic reflex testing was performed using the Testworks3 System (WR Medical, Minneapolis, MN, USA) or the ANX3.0 platform (ANSAR Medical Technologies, Philadelphia, PA, USA) using standard testing and interpretation protocols [15]. Patients were advised to hold medications that may interfere with the interpretation of results, such as beta-blockers, anticholinergics, and adrenergic antagonists, for 48 h prior to testing. The patients presented to the autonomic laboratory and were proctored through the four procedures that comprise the autonomic testing protocol: heart rate deep breathing test, Valsalva maneuver, tilt-table test, and sudomotor testing (Additional Table 1 in Additional File 1). Sudomotor testing assesses the integrity of post-ganglionic peripheral sympathetic nerves that control sweating.

A Composite Autonomic Severity Score (CASS) was assigned to each patient based on their autonomic function [15]. It is the summation of three subdomains: cardiovagal, adrenergic, and sudomotor scores, which are each derived from components of the four autonomic tests. A higher score correlates with a higher degree of autonomic impairment.

### Statistical analysis

Results are presented as mean, standard deviation (SD), and range.

## Results

### Patient demographics, cancer characteristics, and cancer-directed treatments

Of approximately 282 total patients in the cardio-oncology program, 24 patients meeting study inclusion criteria were included (Table 1). The 24 patients were referred for suspected cardiovascular autonomic dysfunction based on the presence of typical symptoms or vital sign abnormalities without a definitive alternative cause. Eight patients were female (33.3%), and the mean age at time of autonomic testing was 51.3 years (SD ± 14.7, range 25–76). The average duration from cancer diagnosis to autonomic testing was 10.3 years (SD ± 12.7, range 0.6–44.1). The reasons for referral for autonomic testing were dizziness (*n* = 13, 54.2%), tachycardia (*n* = 13, 54.2%), palpitations (*n* = 12, 50%), syncope (*n* = 6, 25%), and dyspnea on exertion (*n* = 7, 29.2%), with patients having multiple overlapping symptoms in the absence of significant cardiopulmonary disease. The majority of patients had Hodgkin’s lymphoma (*n* = 9, 37.5%) or acute leukemia (*n* = 5, 20.8%) (Table 2). In terms of anatomic location, most tumors were located in the blood and bone marrow (*n* = 9, 38%) and mediastinum (*n* = 9, 38%).

**Table 1:** Baseline patient characteristics

Baseline Patient Characteristics	
*Demographics*	*n* = 24
Female (n)	33.3% (8)
Age at time of autonomic testing, mean years ± SD (range)	51.3 ± 14.7 (25–76)
Age at time of cancer diagnosis, mean years ± SD (range)	40.9 ± 19.8 (5–70)
Time since cancer diagnosis to autonomic reflex testing, mean years ± SD (range)	10.3 ± 12.7 (0.6–44.1)
Time since remission to autonomic testing, mean years ± SD (range)	8.0 ± 12.3 (− 0.07–44.0)
*Referral Symptom*	*n* = 24
Dizziness (n)	54.2% (13)
Dyspnea on Exertion (n)	29.2% (7)
Palpitations (n)	50% (12)
Syncope (n)	25% (6)
Tachycardia (n)	54.2% (13)

*SD* Standard deviation

**Table 2:** Types and location of diagnosed malignancy

Cancer characteristics	
*Primary Malignancy Type*	*n* = 24
Acute Leukemia (n)	20.8% (5)
Adenocarcinoma (n)	8.3% (2)
Aplastic Anemia (n)	4.2% (1)
Hodgkin’s Lymphoma (n)	37.5% (9)
Lobular Breast Carcinoma (n)	12.5% (3)
Multiple Myeloma (n)	8.3% (2)
Nodal Marginal Zone B-cell Lymphoma (n)	4.2% (1)
Papillary Thyroid Carcinoma (n)	8.3% (2)
Rhabdomyosarcoma (n)	4.2% (1)
Squamous Cell Carcinoma (n)	4.2% (1)
*Location of Majority of Disease*	*n* = 24
Breast (n)	17% (4)
Blood and Bone Marrow (n)	38% (9)
Colon (n)	4% (1)
Mediastinum (n)	38% (9)
Neck (n)	12.5% (3)
Skull (n)	4% (1)
Stomach (n)	4% (1)

Most patients received radiation therapy to the thorax (*n* = 10, 66.7%) and neck (*n* = 8, 53.3%) (Table 3). The most common chemotherapy agents used were alkylating agent (*n* = 16, 66.7%), vinca alkaloids (*n* = 13, 54.2%), and anthracyclines (*n* = 13, 54.2%). Eight patients (33.3%) received hematopoietic stem cell transplantation. Graft versus host disease (GVHD) was present in five of these patients at the time of testing (62.5%). The mean time from hematopoietic stem cell transplant to ARS was 58.1 months (SD ± 82.3, range 1.0–237.1).

**Table 3:** Tumor-directed therapeutic interventions

Tumor-directed therapeutic interventions	
*Radiation Therapy by Location*	*n* = 15
Head (n)	13.3% (2)
Neck (n)	53.3% (8)
Thorax (n)	66.7% (10)
Abdomen (n)	6.7% (1)
Whole Body (n)	13.3% (2)
*Most Common Chemotherapies by Mechanism of Action*	*n* = 24
Anthracycline (n)	54.2% (13)
Anti-Metabolite (n)	37.5% (9)
Alkylating Agent (n)	66.7% (16)
Bleomycin (n)	25% (6)
Microtubule Inhibitor (n)	66.7% (16)
Platinum-Based DNA Crosslinking (n)	20.8% (5)

*SD* Standard deviation, *GVHD* Graft versus host disease

### Autonomic function in cancer survivors

**Fig. 1:**
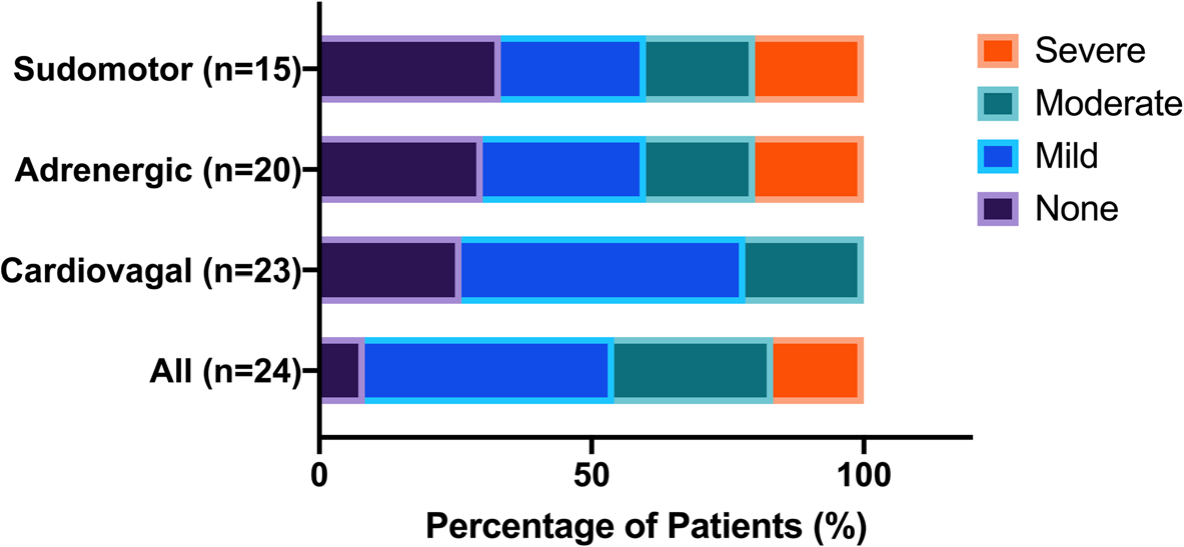
Composite Autonomic Severity Score (CASS) in Cancer Survivors Diagnosed with Autonomic Dysfunction. The severity and distribution of autonomic dysfunction is quantified using the CASS to assess the sudomotor, adrenergic, and cardiovagal systems. The presence of impairment in any system is reflected by "All"

**Fig. 2:**
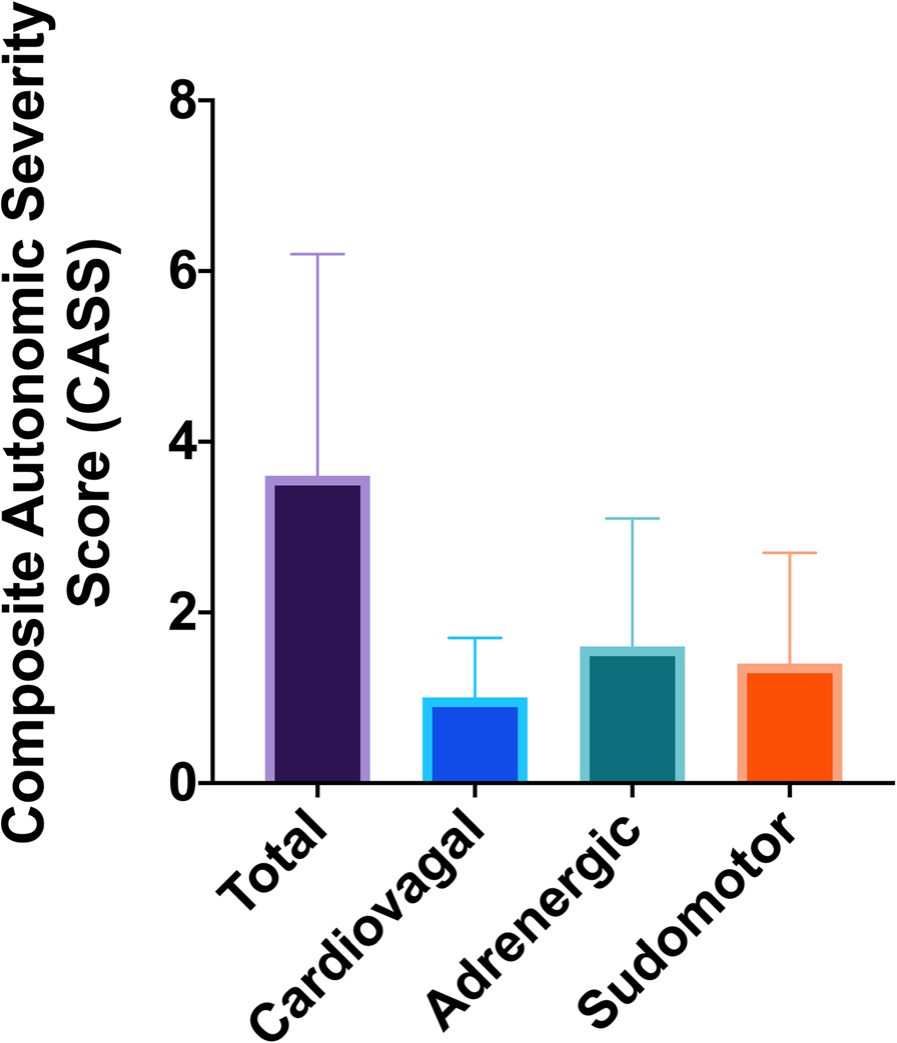
Severity of Autonomic Impairment in Cancer Survivors. Using the composite autonomic severity score (CASS), the total score, and scores for each tested component (cardiovagal, adrenergic, and sudomotor) are shown. For total CASS; normal = 0; mild, moderate, and severe impairment are 1–3, 4–6, & 7–9 respectively. Each component (cardiovagal, adrenergic, and sudomotor) is graded on a scale of 0–3, where 0 indicates no impairment, and 1, 2, & 3 indicated mild, moderate, and severe impairment, respectively

Twenty-four patients underwent autonomic reflex testing (Fig. 1). Of these patients, 22 (92%) had evidence of autonomic impairment on autonomic reflex testing. On average, patients demonstrated mild-to-moderate generalized autonomic impairment (CASS 3.6 SD ± 2.6, range 0–9) and four patients (16.7%) had severe generalized autonomic dysfunction. All three domains (cardiovagal, adrenergic, and sudomotor) demonstrated impairment. However, the degree of dysfunction was most pronounced in the adrenergic system (Fig. 2), while more patients had impairment in cardiovagal function albeit minor. The results of individual autonomic tests are reported in Additional Table 2 (see Additional File 1).

Patients were diagnosed with orthostatic hypotension (*n* = 12, 50%), inappropriate sinus tachycardia (IST) (*n* = 5, 20.8%), and postural orthostatic tachycardia syndrome (POTS) (*n* = 3, 12.5%). Six (25%) of the patients had a diagnosis of heart failure, with three having previously reduced LVEF and three having preserved LVEF. The majority of patients demonstrated subjective response to pharmacologic autonomic treatments including ivabradine, metoprolol, midodrine, and steroids (hydrocortisone and fludrocortisone).

### Cardiac evaluation

On transthoracic echocardiography (TTE), the mean LVEF at time of the autonomic reflex study was 61.2% (SD ± 6.9, range 42.5–75). Three patients had previously reduced LVEF, but all had recovered by the time of autonomic reflex testing. Twenty patients underwent ambulatory cardiac rhythm monitoring. The mean of the average heart rate on ambulatory rhythm monitoring was 90.7 BPM (SD ± 13.5, range 65–117). Fifteen patients completed exercise stress testing. None of the 17 patients had evidence of ischemia with exercise, though two patients (13.3%) did not achieve adequate workloads and had non-diagnostic tests. Cardiodiagnostic results are reported in Additional Table 3 (see Additional File 1).

## Discussion

The main findings of the present study are: 1) nearly all cancer survivors referred for suspicion of autonomic dysfunction tested positive; 2) dysfunction was demonstrated in all domains of the autonomic nervous system, with particular derangement in the sympathetic system; and 3) patients showed subjective and/or objective symptom response to treatment. These data suggest that cardiovascular autonomic dysfunction may be more prevalent than previously recognized in cancer survivors, and should be screened for, tested, and treated when appropriate.

The results of the autonomic reflex testing suggest that cardiovascular autonomic dysfunction may result predominantly from a systemic process, such as chemotherapy or paraneoplastic syndrome, with a different etiology than sensory peripheral neuropathy caused by many chemotherapy agents. Hematologic disorders, hematopoietic stem cell transplantation, poor functional status, vinca alkaloids, alkylating agents, anthracyclines, chest radiation, and neck radiation were associated with autonomic dysfunction. The majority of patients were diagnosed with orthostatic hypotension, POTS, or IST and had subjective improvement with personalized treatment. To the best of our knowledge, this is the first study to report these findings in cancer survivors with suspected autonomic dysfunction. Clinicians caring for these patients should have a low threshold to refer for autonomic evaluation.

### Autonomic function

Cardiovascular autonomic dysfunction is commonly seen in patients with neurodegenerative disorders and diabetes mellitus [16]. In diabetes mellitus, the mechanism is thought to be due to metabolic and ischemic damage to both the sympathetic and parasympathetic systems [17]. In Parkinson’s disease and multiple system atrophy, the underlying mechanism of autonomic impairment is thought to be due to sympathetic denervation of the heart and vasculature [18, 19]. The pathologic mechanism of cardiovascular autonomic dysfunction in cancer survivors is poorly understood [5]. In this study, both the parasympathetic and sympathetic arms demonstrated dysfunction, with a greater degree of impairment in the sympathetic nervous system. This phenotype is similar to that seen in multiple system atrophy. Diabetes and Parkinson’s disease tend to have more proportionate distribution of impairment between the sympathetic and parasympathetic arms [20–22].

The patients in this study also had derangements on sudomotor testing, which assesses the integrity of post-ganglionic sympathetic nerves. Moreover, the degree of sudomotor impairment tended to increase with increasing levels of sympathetic and parasympathetic dysfunction. This suggests that autonomic dysfunction in cancer survivors may result from a diffuse process, such as chemotherapy or paraneoplastic effects, as opposed to a local process, such as radiation therapy, tumor invasion, or inflammation surrounding the malignancy.

### Patient characteristics

The heterogeneity in age and time to autonomic testing may reflect a referral bias due to the non-specific nature of these symptoms [5, 12]. It is plausible that patients who were sent for autonomic reflex testing later in their cancer survivorship were symptomatic for an extended period of time. In this case, testing may have been delayed due to low suspicion for autonomic etiology, accessibility issues, or lack of familiarity with autonomic reflex testing. Another explanation is that these findings represent multiple mechanisms of neurotoxicity that vary in time from original insult to clinical presentation. Moreover, patients with obvious, severe autonomic dysfunction or subtle autonomic dysfunction may not have been referred for autonomic reflex testing due to lack of perceived diagnostic utility or low clinical suspicion for autonomic etiology, respectively.

The majority of patients (75%) had hematologic disorders, which was disproportionately higher than the 9.5% estimated prevalence among cancer survivors in the United States [23]. Many of the chemotherapy regimens used to treat these disorders, such as CHOP (cyclophosphamide, doxorubicin, vincristine, prednisone) or ABVD (doxorubicin, bleomycin, vinblastine, dacarbazine), include anthracyclines and vinca alkaloids. Given the diffuse pattern of autonomic impairment seen on reflex testing, the association between hematologic malignancies and autonomic dysfunction may be due to paraneoplastic effects of the malignancy or the chemotherapies used in those patients, as opposed to a more localized process such as direct tumor invasion.

Eight of the patients received hematopoietic stem cell transplantation. Deuring et al. found that patients who received hematopoietic stem cell transplantation had higher degree of autonomic dysfunction compared to controls [24]. This may suggest autonomic impairment was either due to graft versus host disease or effects of the myeloablative regimen. Most patients had malignancies either in the neck or thorax or had received radiation treatments in those areas. While the results of the autonomic reflex testing suggest a more diffuse neuropathy, it is possible that local tumor-related effects had some effect given that the autonomic baroreflex pathways are located the neck and thorax as well.

The effects of chemoradiation on peripheral sensory nerves are well established [25]. Given the similarities in myelination between peripheral sensory and autonomic nerves, these mechanisms have been extrapolated to be possible explanations for autonomic dysfunction [25]. The most common chemotherapy agents in this study were vinca alkaloids, alkylating agents, and anthracyclines. There is literature suggesting associations between these agents and autonomic impairment. Microtubule inhibitors, such as vinca alkaloids, are a well-known cause of peripheral sensory neuropathy [25]. The effect on the autonomic system was evaluated by a Roca et al., who found that vinca alkaloids treatment altered orthostatic blood pressure and heart rate with deep breathing [26]. While alkylating agents are not commonly associated with neuropathy, a study by Dobrek et al. demonstrated a relationship between alkylating agents and alterations in heart rate modulation [27]. The two patients in our study who received alkylating agents and did not receive microtubule inhibitors, anthracyclines, or platinum-based agents had severe generalized autonomic impairment most prominent in the adrenergic and sudomotor systems. Anthracyclines have well-established association with cardiotoxicity, but not necessarily with neuropathy. Anthracyclines have been associated with abnormalities in heart rate variability [28]. In patients with diabetes, there is some evidence that cardiac autonomic dysfunction is associated with non-ischemic cardiomyopathy [29]. The one patient who received anthracyclines, but did not received microtubules or alkylating agents, had moderate autonomic dysfunction with severe adrenergic impairment.

The association between chemotherapy agents, such as anthracyclines and alkylating agents, that are not traditionally associated with neuropathy and cardiovascular autonomic dysfunction suggest that autonomic impairment in cancer survivors may be caused by a different mechanism than the sensory peripheral neuropathy seen with many chemotherapy agents. Moreover, given the number of patients in this study that had cardiomyopathy or received anthracyclines, it is possible that autonomic dysfunction is a precursor to, or at the very least, associated with cardiomyopathy [4].

### Clinical implications

Cardiovascular autonomic dysfunction in cancer survivors is a poorly understood condition that carries significant morbidity and is associated with an increase all-cause mortality [13]. In this study, most patients had either orthostatic hypotension, POTS, or IST, which are recognized by the Heart Rhythm Society as autonomic diseases [30]. Given our limited knowledge on cardiovascular autonomic dysfunction in cancer survivors, there are currently no published guidelines on treatment options [31]. Evidence-based pharmacologic treatment strategies for autonomic dysfunction are largely based on studies of patients with neurodegenerative diseases, diabetes mellitus, and infiltrative diseases [32]. The effectiveness of treatment is generally varied and often poor. In this cohort, the results of autonomic reflex testing were used to tailor personalized, autonomic treatment to each patient. The majority of patients had subjective improvement in treatments, which further demonstrates the utility of this tool.

This study also exemplifies the role of a cardio-oncology program in identifying, preventing, and managing the unique cardiovascular comorbities, such as cardiovascular autonomic dysfunction, faced by this population to improve their overall quality of life. Physicians caring for these patients should have a low threshold to refer for autonomic evaluation, particularly for patients with hematologic malignancies, tumor burden in the neck or chest, cardiomyopathy, or those who have received vinca alkaloids, alkylating agents, anthracyclines, or hematopoietic stem cell transplantation. Autonomic reflex testing may suggest personalized treatment options to improve outcomes and provide an objective measure to assess response to treatment and progression of disease.

### Limitations

This study has several limitations. First, as this study was a retrospective, descriptive case series, statistical hypothesis testing and control groups were not used given the small sample size and large number of variables (cancer types, chemotherapy agents, etc.). There is a selection bias as only symptomatic patients were referred for autonomic reflex testing, which represents a select subset of the total cardio-oncology patient population at this institution. However, given the significantly limited data that exists in this population, particularly among symptomatic patients rather than patient with incidentally found autonomic dysfunction, we believe this information provides useful data for the field. Moreover, there is also a referral bias as the proportion of different malignancies managed and the specific treatment regimens used for different cancers vary per institution. Additionally, as cancer treatments were composed of multi-drug regimens, individual associations were difficult to identify. Furthermore, given the difficulty in determining equipotency between doses and routes of administration between different chemotherapeutic agents, medication use was classified by the presents of administration rather than by cumulative dosing. Autonomic treatments were personalized, which led to a lack of standardization with regard to treatment modality. However, given the paucity of data on this topic, we believe this study provides novel insights into the diagnosis of and management of dysautonomia associated with cancer treatment.

## Conclusion

Cardiovascular autonomic dysfunction is an emerging, but poorly understood topic in cancer survivors. In this cohort study, cancer-directed treatments were associated with cardiovascular autonomic dysfunction. Large prospective studies focusing on confirming these associations and determining the efficacy of individual therapies based on dysautonomic mechanisms and certain cancer treatments are warranted. This will likely improve quality of life metrics and potentially attenuate short and long term comorbidity in this unique and poorly understood patient population.

## Supplementary information

**Additional file 1: Additional Table 1.** Autonomic Reflex Testing Interpretation. **Additional Table 2.** Autonomic Results by Individual Tests. **Additional Table 3.** Cardiodiagnostic Testing.

## Data Availability

The datasets used and/or analyzed during the current study are available from the corresponding author on reasonable request.

## Abbreviations

ABVD: Doxorubicin, bleomycin, vinblastine, dacarbazine
afib: Atrial fibrillation
ARS: Autonomic reflex study
avg: Average
BP: Blood pressure
BPM: Beats per minute
CASS: Composite Autonomic Severity Score
CHOP: Cyclophosphamide, doxorubicin, vincristine, prednisone
DBP: Diastolic blood pressure
GVHD: Graft versus host disease
HR: Heart rate
HRDB: Heart rate deep breathing
HSCT: Hematopoietic stem cell transplantation
IST: Inappropriate sinus tachycardia
JoVE: Journal of Visualized Experiments
LVEF: Left ventricular ejection fraction
MAP: Mean arterial blood pressure
max: Maximum
METs: Metabolic equivalents
min: Minimum
PASP: Pulmonary artery systolic pressure
POTS: Postural orthostatic tachycardia syndrome
RSA: Respiratory sinus arrhythmia
SBP: Systolic blood pressure
SD: Standard deviation
secs: Seconds
SVT: Supraventricular tachycardia
TTE: Transthoracic echocardiography
