# Supplementary material for: Quantitative assessment of cardiovascular autonomic impairment in cancer survivors: a single center case series
Source: Cardiooncology. 2020 Jul 28;6:11. doi: 10.1186/s40959-020-00065-9 (PMC7388471; doi:10.1186/s40959-020-00065-9)
Supplement: Supplementary file 1 — Additional file 1: Additional Table 1. Autonomic Reflex Testing Interpretation. Additional Table 2. Autonomic Results by Individual Tests. Additional Table 3. Cardiodiagnostic Testing. [file 40959_2020_65_MOESM1_ESM.docx]

**Additional File 1**

Additional Table 1. Autonomic Reflex Testing Interpretation. Legend: RSA, respiratory sinus arrhythmia; BPM, beats per minute; HR, heart rate; min, minimum; max, maximum; secs, seconds; MAP, mean arterial blood pressure; BP, blood pressure; SBP, systolic blood pressure; DBP, diastolic blood pressure; POTS, postural orthostatic tachycardia syndrome. Adapted from: Novak P. Quantitative autonomic testing. JoVE (Journal of Visualized Experiments) 2011:e2502

| **Autonomic Reflex Testing Interpretation** | |
| --- | --- |
| Heart rate deep breathing | Mean RSA = average (HR at end expiration - HR at end inspiration) after each cycle  Mean RSA < the age-adjusted min normal RSA was indicative of parasympathetic dysfunction |
| Valsalva maneuver | Valsalva ratio = Max HR/Min HR during the maneuver. Valsalva ratio < age-and-sex-adjusted min normal Valsalva ratio was indicative of parasympathetic dysfunction |
|  | Late phase 2 recovery: Present if peak MAP during late phase 2 ≥ baseline MAP Absence of the late phase two recovery was indicative of sympathetic dysfunction |
|  | Phase 4 overshoot: Present if MAP at phase 4 ≥ baseline MAP Absence of phase four overshoot was indicative of sympathetic dysfunction. |
|  | Phase 4 SBP recovery time: Normal if Phase 4 SBP ≥ baseline SBP within 4 second Prolonged SBP recovery time was indicative of sympathetic dysfunction |
| Tilt table test | Orthostatic hypotension: SBP fell by ≥ 20 mmHg from baseline SBP or the DBP falls by ≥ 10 mmHg from baseline DBP |
|  | POTS: HR increased by ≥ 30 BPM from baseline HR in the absence of criteria for orthostatic hypotension |
| Sudomotor test | Abnormal Sweat Production: Each site was determined to be abnormal if the total volume of sweat production was outside of age-and-sex adjusted normal values |
|  | Length-dependent abnormality: Present if there was a reduction of the distal sweat volume of more than 1/3 of the proximal value, with respect to anatomic location. |

Additional Table 2. Autonomic Results by Individual Tests.

| **Autonomic Results by Individual Tests** | | |
| --- | --- | --- |
| *Sudomotor* | n=15 | |
| Normal |  | 66.7% (10) |
| Abnormal |  | 33.3% (5) |
| *Heart Rate Deep Breathing Test* |  | n=23 |
| Below Normal |  | 39.1% (9) |
| Normal |  | 56.5% (13) |
| Above Normal |  | 4.4% (1) |
| *Tilt Table* |  | n=21 |
| Normal |  | 38.1% (8) |
| Orthostatic Hypotension |  | 47.6% (10) |
| Orthostatic Tachycardia |  | 14.3% (3) |
| *Valsalva Test* |  |  |
| *Valsalva Ratio* |  | n=23 |
| Normal |  | 30.4% (7) |
| Below Normal |  | 69.6% (16) |
| *Late Phase 2 Recovery* |  | n=18 |
| Present |  | 38.9% (7) |
| Absent |  | 61.1% (11) |
| *Phase 4 Recovery Time* |  | n=18 |
| Normal |  | 88.9% (16) |
| Prolonged |  | 11.1% (2) |
| *Phase 4 Overshoot* |  | n=18 |
| Present |  | 83.3% (15) |
| Absent |  | 11.1% (2) |
| Prolonged |  | 5.6% (1) |

Additional Table 3. Cardiodiagnostic Testing. *Reduced LVEF defined as <50%. **Abnormal strain values were based on the age-and-gender adjusted normative values of the software used for interpretation. Legend: ARS, autonomic reflex study; SD, standard deviation; LVEF, left ventricular ejection fracture; PASP, pulmonary artery systolic pressure; BPM, beats per minute; HR, heart rate; avg, average; min, minimum; max, maximum; METs, metabolic equivalents; SBP, systolic blood pressure; DBP, diastolic blood pressure.

| **Cardiodiagnostics** | |
| --- | --- |
| *Transthoracic Echocardiography* | n=24 |
| LVEF closest to ARS mean % ± SD (range) | 61.2 ± 6.9 (42.5-75) |
| LVEF lowest recorded at any time, mean % ± SD (range) | 55 ± 8.6 (27.5-67.5) |
| Reduced LVEF* | 8.3% (2) |
| Recovered LVEF at time of autonomic reflex study | 100% (2) |
| PASP, mean mmHg ± SD (range) | 25.4 ± 6 (16-35) |
| Global Longitudinal Strain** | n=13 |
| Normal (n) | 84.6% (11) |
| Abnormal (n) | 15.4% (2) |
| *Ambulatory Cardiac Rhythm Monitor* | n=20 |
| Predominant Underlying Rhythm |  |
| Sinus Rhythm (n) | 80% (16) |
| Sinus Tachycardia (n) | 20% (4) |
| Avg HR, mean BPM ± SD (range) | 90.7 ± 13.5 (65-117) |
| Min HR, mean BPM ± SD (range) | 60.5 ± 20.1 (42-130) |
| Max HR, mean BPM ± SD (range) | 171.4 ± 33 (131-250) |
| Range, mean BPM ± SD (range) | 110.8 ± 42.3 (25-199) |
| *Exercise Stress Test* | n=17 |
| Exercise Capacity, mean METs ± SD (range) (n=15) | 6.6 ± 2.9 (2-13.4) |
| Subjective Exercise Tolerance | n=17 |
| Poor (n) | 58.8% (10) |
| Fair (n) | 29.4% (5) |
| Good (n) | 5.9% (1) |
| Excellent (n) | 5.9% (1) |
| Inducible Ischemia | n=15 |
| No (n) | 86.7% (13) |
| Non-Diagnostic (n) | 13.3% (2) |
| Resting HR, mean BPM ± SD (range) | 99.9 ± 20.3 (64-152) |
| Resting HR/Age-Adjusted Max Predicted HR, mean % ± SD (range) | 0.59 ± 0.09 (0.43-0.79) |
| Peak HR/Age-Adjusted Max Predicted HR, mean % ± SD (range) | 0.89 ± 0.13 (0.65-1.09) |
| Blood Pressure Response to Stress | n=15 |
| Blunted (n) | 6.7% (1) |
| Normal (n) | 86.7% (13) |
| Hypertensive (n) | 6.7% (1) |
| Resting SBP, mean mmHg ± SD (range) | 122 ± 17.4 (86-153) |
| Resting DBP, mean mmHg ± SD (range) | 76.4 ± 10.7 (49-90) |
| Resting LVEF, mean % ± SD (range) | 57.5 ± 4.7 (47.5-62.5) |
| Peak HR, mean BPM ± SD (range) | 150.5 ± 26.4 (101-196) |
| Peak SBP, mean mmHg ± SD (range) | 155.4 ± 30 (99-225) |
| Peak DBP, mean mmHg ± SD (range) | 70.9 ± 9.4 (57-86) |
| Stress LVEF, mean % ± SD (range) | 70 ± 4.7 (62.5-75) |
